# Psychoactive Substance Use Among Middle-Aged and Older Adults With Visual Impairment

**DOI:** 10.1093/geroni/igab046.3523

**Published:** 2021-12-17

**Authors:** Francisco Lopez, Jason Leddy, Benjamin Han, Joseph Palamar

**Affiliations:** 1 UCSD MADURA R25 Advancing Diversity in Aging Research Program, San Diego, California, United States; 2 UC San Diego, La Jolla, California, United States; 3 New York University, New York, New York, United States

## Abstract

Older adults with visual impairment may be at risk for developing substance use disorder (SUD) as psychoactive substance use is often used to cope with the stressors of vision loss. This study estimates the national prevalence and risk of psychoactive substance use among older adults with visual impairment. We analyzed data of respondents age ≥50 from the 2015-2019 National Survey on Drug Use and Health, an annual cross-sectional survey of a nationally representative sample of non-institutionalized individuals in the U.S. (N=43,886). We estimated and compared prevalence of past-year use of cannabis, cocaine, misuse of prescription opioids, sedatives, stimulants/tranquilizers, alcohol use disorder (AUD), any SUD, and nicotine dependence between adults with visual impairment to those without. Comparisons were conducted using chi-square and we used multivariable generalized linear models using Poisson and log link to estimate adjusted prevalence ratios (aPRs) for adults with visual impairment relative to those without, controlling for demographics and diagnosis of ≥2 chronic diseases. An estimated 6.1% experienced visual impairment. Those with visual impairment had higher prevalence of AUD, nicotine dependence, misuse of prescription opioids, tranquilizers, and stimulants, and SUDs. In adjusted analyses, vision-impaired adults had higher risk of AUD (aPR=1.71, 95% CI: 1.40-2.09), nicotine dependence (aPR =1.53, 95% CI:1.35-1.73), opioid misuse (aPR =1.54, 95% CI:1.26-1.90), and SUD (aPR=1.67, 95% CI:1.40-2.01). Psychoactive substance use adds unique health risks for older adults with vision loss, who may suffer significant psychological stress and loss of independence. Screening for substance use among all older adults with visual impairment should be considered.

